# Neurocysticercosis: Current Perspectives on Diagnosis and Management

**DOI:** 10.3389/fvets.2021.615703

**Published:** 2021-05-10

**Authors:** Caitlin Butala, T. M. Brook, Ayodele O. Majekodunmi, Susan Christina Welburn

**Affiliations:** ^1^Zhejiang University-University of Edinburgh Institute, Zhejiang University School of Medicine, Zhejiang University, Haining, China; ^2^Infection Medicine, Deanery of Biomedical Sciences, Edinburgh Medical School, College of Medicine & Veterinary Medicine, The University of Edinburgh, Edinburgh, United Kingdom

**Keywords:** neurocysticercosis, cysticercosis, epilepsy, mass drug administration, anthelmintics, drug resistance, *Taenia solium*, taeniasis

## Abstract

Porcine cysticercosis, human taeniasis, and (neuro)cysticercosis are endemic in many low- and middle-income countries (LMIC) where they present a significant health burden to affected communities. Neurocysticercosis (NCC) is one of the leading causes of human epilepsy in many hyperendemic regions in Latin America, Asia, and sub-Saharan Africa. The World Health Organization (WHO) reports an estimated 2.5–8.3 million cases of NCC annually with a disability-adjusted life year (DALY) burden of 2.8 million, but as for all neglected tropical diseases (NTDs), these values are likely to be underestimated. Diagnosis of NCC is complex and most accurately diagnosed using clinical neuroimaging that is unavailable in most hyperendemic regions in LMIC. On January 28, 2021, WHO will launch its road map for the NTDs' “Ending the neglect to attain the Sustainable Development Goals: a road map for neglected tropical diseases 2021–2030.” Taeniasis/cysticercosis is targeted for control success considered as steady increase in the number of countries with intensified control in hyperendemic areas [increasing from 2 (3%) in 2020 to 4 (6%) in 2023, to 9 (14%) by 2025, and to 17 (27%) by 2030]. Cross-cutting targets that include 100% access to at least basic water supply, sanitation, and hygiene in areas endemic for NTDs and 75% integrated treatment coverage for preventative chemotherapy will additionally impact on the taeniasis/cysticercosis/NCC complex. With no vaccine available for humans, prevention of infection depends on communication to the public of the life cycle of a complex zoonosis to promote behavior change, underpinned by practical control measures including treatment of human taeniasis and (neuro)cysticercosis with albendazole and praziquantel [widely used as part of the mass drug administration (MDA) deworming programs], surgery where appropriate, and effective vaccination and deworming for pigs supported by meat inspection. Here, we review recent advances in tools and implementation for *Taenia solium* taeniasis/(neuro)cysticercosis (TSTC) control and milestones on the onward path to elimination.

## Introduction

Taeniasis and (neuro)cysticercosis are caused by the cestode *Taenia solium* or pork tapeworm. *T. solium* is a multi-host parasite with a complex zoonotic transmission cycle, circulating between the intermediate pig host and the definitive or accidental intermediate human host (1, 2). *T. solium* infection arises from ingestion of contaminated food or water and ingestion of raw or undercooked pork and may result in taeniasis (caused by the adult tapeworm living in the small intestine) and/or cysticercosis or neurocysticercosis [NCC; caused by invasion of the larvae into the central nervous system (CNS)] in humans.

When humans consume pork containing live *T. solium* cysts, the cysticercus develops into a mature tapeworm in the human intestine, shedding eggs that are expelled in human feces.

Cysticercosis develops when, following ingestion of *T. solium* eggs, *T. solium* larvae migrate and become encysted, typically in the muscle tissue of the host. Pigs can harbor thousands of cysts. When *T. solium* cysticerci develop in the human brain, the condition is defined as NCC. NCC is the most common parasitic disease of the CNS in humans affecting between 2.5 and 8.3 million people annually, accounting for a global burden of 2.8 million disability-adjusted life years (DALYs) (1, 2). NCC is a major clinical consequence of *T. solium* infection and the dominant cause of global preventable epilepsy associated with morbidity and mortality from epileptic seizures and epilepsy related death; where *T. solium* is endemic, 30% of epilepsy cases are estimated to be caused by NCC (3, 4).

Taeniasis can cause abdominal pain, nausea, and diarrhea, although it is often asymptomatic, at around 8 weeks post-ingestion with symptoms persisting until treatment with anthelmintic drugs or for around 2–3 years (the lifespan of the adult tapeworm) if untreated (5, 6). Many carriers of *T. solium* (taeniasis and cysticercosis) are asymptomatic and become long-term carriers of infection through self-reinfection and re-infection from others within the household (6). *T. solium* infection in pigs can be detected by meat inspection by visual inspection of cut meat and by lingual examination of the live animal; this, however, has low sensitivity as cysts can be missed.

As for most of the neglected zoonoses, under-diagnosis and under-reporting of cysticercosis and NCC result in underestimation of case numbers and global burden (1, 7, 8). NCC is endemic in low- and middle-income countries (LMIC), where sanitation and clean water are substandard, and in communities where pig-keeping is an integral part of the local economy and often promoted as a route out of poverty (8, 9). NCC is becoming more prevalent in developed economies with increased immigration from regions endemic for *T. solium* (10).

## Neurocysticercosis

In cases of NCC, *T. solium* larvae are found either in the brain tissue (parenchymal NCC) or in the intraventricular and subarachnoid spaces of the brain and spinal cord where the cerebrospinal fluid (CSF) circulates (extraparenchymal NCC) resulting in different clinical manifestations and prognoses. Parenchymal NCC manifests with seizures and headaches with psychiatric symptoms being rare and generally has a better prognosis since seizures tend to respond well to anti-seizure drug therapy (11–13). Extraparenchymal NCC may result in increased intracranial pressure and hydrocephalus, and patients show poorer prognosis, in part due to the growth (increase in size) of cysts in the subarachnoid space prior to symptoms becoming apparent and from late diagnosis (14).

The clinical presentation of NCC is similar to a wide range of neurological conditions making clinical diagnosis, especially in low-income country settings, difficult. Depending on the number, size, stage, and location of the cysts and the immune response of an individual patient, NCC presentation can vary from being asymptomatic to sudden death. A definitive clinical diagnosis is only made by visualization of cysts or larvae in the brain tissue *via* neuroimaging (15–17); in some cases, intracranial calcification of cysts is the only evidence of the disease (5).

Studies relating infection to mortality are rare. The limited number of hospital-based studies reporting deaths, in general, reports mortality from extraparenchymal NCC. In Brazil, endemic for *T. solium*, 1,570 NCC deaths were reported between 1985 and 2011, whereas in the United States (non-endemic), 221 NCC deaths were reported between 1985 and 2011 (14, 18, 19). These numbers represent deaths where NCC was considered the direct or an associated cause of death. In the absence of imaging or autopsy data, NCC-associated deaths are invariably under-reported.

## Diagnosis

Established methods for NCC diagnosis include a detailed clinical examination, serological testing, and neuroimaging. Each method has its benefits and drawbacks, some being more successful at diagnosing NCC infection at different stages (cysts, calcified cysts). Definitive classifications have been provided by Del Brutto et al. (20), revised in 2017 (Table 1), and Carpio et al. (Table 2) (20–22).

**Table 1:** Diagnostic criteria [derived from (20, 21)].

**Definitive** • Histological demonstration of the parasite from biopsy of a brain or spinal cord lesion • Evidence of cystic lesions showing the scolex on neuroimaging studies • Direct visualization of subretinal parasites by fundoscopic examination Neuroimaging criteria: Major neuroimaging criteria: • Multilobulated cystic lesions in the subarachnoid space • Typical parenchymal brain calcifications Confirmative neuroimaging criteria • Resolution of cystic lesions after cysticidal drug therapy • Spontaneous resolution of single small enhancing lesions • Migration of ventricular cysts documented on sequential neuroimaging studies Minor neuroimaging criteria • Obstructive hydrocephalus (symmetric or asymmetric) or abnormal enhancement of basal leptomeninges
**Clinical/exposure criteria** Major • Evidence of lesions highly suggestive of neurocysticercosis on neuroimaging studies • Positive serum immunoblot for the detection of anticysticeral antibodies or cysticeral antigens by well-standardized immunological tests • Resolution of intracranial cystic lesions after therapy with albendazole or praziquantel • Spontaneous resolution of single small enhancing lesions • Cysticercosis outside the central nervous system • Evidence of contact with T. solium infection
Minor • Evidence of lesions compatible with neurocysticercosis on neuroimaging • Presence of clinical manifestations suggestive of neurocysticercosis • Positive CSF ELISA for the detection of anticysticeral antibodies or cysticeral antigens • Evidence of cysticercosis outside the central nervous system • Individuals coming from or living in an area where cysticercosis is endemic
**Epidemiological** • Individuals coming from or living in an area where cysticercosis is endemic • History of travel to disease-endemic areas • Evidence of household contact with T. solium infection
**Degrees of diagnostic certainty** Definitive • Presence of one absolute criterion • Presence of two major plus one minor and one epidemiological criteria • Two major neuroimaging criteria plus any clinical/exposure criteria • One major and one confirmative neuroimaging criteria plus any clinical/exposure criteria • One major criterion plus two clinical/exposure criteria (including at least one major clinical/exposure criterion) together with the exclusion of other pathologies producing similar neuroimaging findings Probable • Presence of one major neuroimaging plus two minor clinical/exposure criteria • Presence of one major plus one minor and one epidemiological criterion • Presence of three minor and one epidemiological criterion • One minor neuroimaging criteria plus at least one major clinical/exposure criteria

*Diagnostic criteria from 2001 (in black) and changes from Del Brutto et al. (21) (in red). Criteria moved or deleted from the original are in blue. CSF, cerebrospinal fluid; ELISA, enzyme-linked immunosorbent assay*.

**Table 2:** Definitive diagnostic criteria for symptomatic neurocysticercosis by Carpio et al. (22).

**Parenchymal neurocysticercosisDefinitive parenchymal neurocysticercosis, one of the following:** 1. Parenchymal cyst with pathological diagnosis 2. Single or multiple active parenchymal cysts, with at least one cyst with scolex on CT or MRI 3. Multiple parenchymal vesicles without scolex associated with at least one of the following: a. Seizures: focal or generalized tonic–clonic b. Positive serum or CSF immunological test (ELISA, EITB) 4. Any combination of the parenchymal cysticercus in different evolutive stages: vesicular with or without scolex, degenerative (colloidal or nodular), and calcified Probable parenchymal neurocysticercosis, one of the following: 1. Single parenchymal calcification or vesicle (without scolex) or degenerating cyst(s), establishing differential diagnoses with other etiologies, associated with at least two of the following: a. Seizures: focal or generalized tonic–clonic b. Subcutaneous or muscle cysts location confirmed by biopsy c. Positive serum or CSF immunological test (ELISA, EITB) d. Plain X-ray films showing “cigar-shaped” calcifications e. Individual who lives or has lived in or has traveled frequently to endemic countries 2. Multiple parenchymal calcifications in an individual who lives or has lived in or has traveled frequently to endemic countries and in whom clinical state excludes other etiologies of calcifications
**Extraparenchymal neurocysticercosis (intraventricular/basal subarachnoid)** Definitive extraparenchymal neurocysticercosis, one of the following: 1. Extraparenchymal cyst with pathological diagnosis 2. One or more extraparenchymal cysts on MRI special sequences with scolex in at least one of them 3. One or more extraparenchymal cysts on MRI special sequences without scolex associated with at least two of the following: a. Hydrocephalus b. Inflammatory CSF c. Positive CSF immunological test (ELISA, EITB) d. Presence of single or multiple calcifications or parenchymal vesicular or degenerative cyst
**Parenchymal and extraparenchymal neurocysticercosis** Combination of the above definitive parenchymal and definitive extraparenchymal criteria

*CT, computed tomography; MRI, magnetic resonance imaging; CSF, cerebrospinal fluid; ELISA, enzyme-linked immunosorbent assay; EITB, enzyme-linked immunoelectrotransfer blot*.

### Serological Diagnostic Tests

Serological methods enable the detection of specific anti-*T. solium* antibodies or *T. solium* antigens in the blood, urine, and CNS (23, 24). Testing for *T. solium*-specific antibodies does not differentiate between an active infection or exposure from a previous infection (24, 25). Enzyme-linked immunoelectrotransfer blot (EITB) identifies specific antibodies to lentil lectin purified glycoprotein (LLGP-EITB) antigens of *T. solium*. In patients with multiple parenchymal cysts, or subarachnoid NCC, EITB has near 100% sensitivity (26, 27); however, in patients with only calcified cysts or single parenchymal lesions, the test reaches only 60–70% sensitivity (25, 27). Enzyme-linked immunosorbent assay (ELISA) detection of *T. solium* antibodies using crude or purified parasitic antigen extracts uses IgG as the target immunoglobulin; however, Ab-ELISAs generally have a lower specificity and sensitivity of EITB (28, 29). Despite this, specific ELISAs are useful in confirming diagnosis and evaluating treatment of extraparenchymal cysts (30). Detection of circulating cysticercus antigens can be done by using monoclonal antibody-based antigen capturing ELISA (Ag-ELISA) (23, 31). These tests only detect the presence of active viable cysts. In combination with antibody-detecting tests, Ag-ELISA can be used to differentiate between live parasite infections and dead larvae in degenerating cysts (23, 31); high antigen levels are associated with extraparenchymal NCC, whereas low or undetectable antigen levels are associated with intraparenchymal NCC (23, 31).

In the absence of neuroimaging, serological tests can assist in making a diagnosis of extraparenchymal NCC or intraparenchymal NCC; this is critical in low-income country settings where neuroimaging is not readily available outside of major hospitals. However, lower-cost diagnostic tools are desperately needed for LMIC for which the infection is endemic. ELISA kits vary from US$5 to US$30 per sample, and cross-react with echinococcosis where the diseases are co-endemic (32, 33). EITB tests range from US$22 to US$100 but can cost as much as US$347 per sample (34).

### Neuroimaging

Neuroimaging is the gold standard for NCC diagnosis, but in many areas endemic for NCC, this technology is either unavailable or prohibitively expensive. Magnetic resonance imaging (MRI) or computed tomography (CT) is used to visualize cysticerci in the CNS, providing evidence of the number of cysts, topography of lesions, stage of evolution of the cyst, and assessment of the level of the host's inflammatory reaction against parasites. Where available, CT scanning is the most common imaging tool used for diagnosis, especially in developing countries; however, CT is less effective than MRI at identifying intraventricular cysts, which comprise up to 22% of all NCC cases (35, 36).

In 2017, Del Brutto et al. revised their diagnostic criteria for NCC to include neuroimaging with a view to eliminating false-positive diagnoses in endemic areas (from serological examinations) and increase diagnosis in non-endemic areas where NCC is often overlooked (21). The revised diagnostic criteria determine that NCC cannot be definitively diagnosed without neuroimaging, and that for a definitive NCC diagnosis, the tapeworm scolex (head) should be visible on the scan (21, 37). However, neuroimaging is unavailable in many endemic areas, training of radiologists for correct interpretations of the scans can be problematic in developing countries, and the high cost of imaging precludes initial and sequential scans.

## Treatment

Treatment options include destroying the cysts using chemotherapy, surgically removing the cysts, and/or application of symptomatic treatment (with or without removal of cysts). Normally, therapy involves the administration of a combination of cysticidal drugs and drugs to alleviate symptoms (38).

### Chemotherapy

The anthelmintic drugs praziquantel and albendazole have been routinely used to control schistosomiasis, cysticercosis, and intestinal nematodes for over 30 years (39). NCC can be treated using albendazole and/or praziquantel, and while there have been changes to recommended dosages, these remain the only drugs available for NCC treatment. Albendazole cannot be taken by pregnant women but can be given in smaller doses to children over the age of 2 (40). Praziquantel can be used by both pregnant women and children and is the preferred treatment for pregnant women (40). Neither are 100% effective due to poor absorption; praziquantel has an oral absorption rate of 80%, whereas albendazole has an oral absorption rate of less than 5% (although this increases to up to 25% if taken with a high-fat meal) (2, 9).

Praziquantel is commonly prescribed at a dosage of 50 mg/kg/day for 10–14 days (6); it is rapidly absorbed (26, 41). Albendazole is typically given as 15 mg/kg/day for 10–14 days (26). In the event of severe disease and for some parenchymal cases, an extended treatment of 30 days of albendazole may be required. In comparative clinical trials, albendazole was equivalent or superior to praziquantel in terms of reduction of live cysticerci (26). Treatment with albendazole at 15 mg/kg/day for 10 days, with 6 mg dose of dexamethasone to reduce inflammation, was shown to reduce the frequency of generalized seizures over 30 months following treatment (2).

Albendazole penetrates the CSF more efficiently than praziquantel (6) and is more effective against extraparenchymal forms and is prescribed more frequently. In some patients, these drugs may exacerbate the symptom of intracranial hypertension with cysticercotic encephalitis (42).

Treating with a combination of the two drugs may be optimal in some cases, since some patients respond better to one drug or the other (2, 43). Garcia and Del Brutto found that in patients with multiple brain cysts, treating with albendazole and praziquantel increased cysticidal effects without potentiating drug-induced side effects (44). Routine dual prescribing may, however, contribute to the risk of development of anthelmintic resistance (AR).

### Complications of Chemotherapy

When a cyst is destroyed by cysticidal drugs, the resulting inflammatory reaction may be pathogenic, appearing acutely as a brain edema or chronically as a gliotic scar (12). To avoid complications from a rise in intracranial pressure, seizures, or epileptic scar, some clinicians argue against using cysticidal drugs and recommend symptomatic treatment (anti-epileptic drugs) and/or surgery to remove the cysts. Anti-epileptic drugs normally adequately control seizures in patients with calcified cysticerci, whereas mannitol can relieve intracranial pressure (12).

Steroids administered together with cysticidal drugs can suppress the inflammatory response associated with the destruction of viable cysts and control edema that is associated with the lesions (1, 12).

### Surgery

Surgery is a recommended treatment for NCC in cases of intraventricular cysts, hydrocephalus, or when the diagnosis is uncertain from neuroimaging (45). Calcified cysts can be removed by minimally invasive neuroendoscopy prior to the administration of cysticidal drugs as the drugs may cause the cysts to rupture and create an inflammatory response to impair removal (21), and/or a ventricular shunt can be inserted to reduce intracranial pressure (45, 46). Limited data exist as to the number of surgeries performed annually to remove the cysts; whether this is due to poor reporting record or a lack of emphasis to report remains unclear.

## The Future for Diagnosis, Treatment, and Prevention

### Diagnosis

Simple, cheap, and effective diagnostic tools are needed to identify infections and at-risk groups and communities. Toribio et al. have demonstrated the viability of extracting *T. solium* DNA in patients' urine, confirmed with positive EITB results for the presence of anti-*T. solium* antibodies in all subarachnoid and patients with viable parenchymal cysts. The sensitivity of the urine test is, however, dependent on infection load, and similar to all serological tests, it cannot determine whether the cysts are present in the CNS or elsewhere in the body (47).

Portable fluorescent sensors that can detect antibodies and enable results to be captured on a mobile device and reviewed later offer significant benefits for diagnosis and surveillance. These tools can enable the identification of hyperendemic areas to target for control. Being able to make a diagnosis while the patient is still in the vicinity and enabling data to be assimilated for prevalence and control studies would support control efforts (48).

A real-time quantitative polymerase chain reaction (qPCR) test to detect the repetitive Tsol13 sequence within the *T. solium* genome has been shown to be highly sensitive and specific for NCC and can be used as a marker for “cure” in the CSF and for the definitive diagnosis of NCC from plasma samples (49). Out of 18 CSF samples taken from patients with active NCC, all were found positive for *T. solium* DNA using TsolR13 qPCR (49).

Advances in neuroimaging will continue to improve the early diagnosis and treatment of NCC. In Mexico, a population study of 155 apparently asymptomatic, healthy patients underwent MRI scanning, and 9.1% were found to have calcified lesions (50). A new Food and Drug Administration (FDA) approved portable MRI machine offers the opportunity to make MRIs more accessible in hospitals and clinics in LMIC (51).

### Treatment

A new delivery system for triclabendazole has been developed for the treatment of trematodes, promoting absorption by encapsulating triclabendazole into nanometer-sized capsules using nanoparticles to increase the drug dissolution rate (52). Similar approaches could be applied to albendazole and praziquantel (52).

A tumor necrosis factor alpha (TNF-α) inhibitor TNF etanercept (ETN) is being trialed to reduce inflammation resulting from TNF-α and other pro-inflammatory cytokines from the administration of cysticidal drugs in NCC patients resulting in fewer symptoms for the patients (12). Anecdotal success has been reported in 16 patients with reductions in corticosteroid usage and decreases in headaches and seizures (53). However, since most patients were taking methotrexate, it is unclear whether ETN alone or used with methotrexate is key to clinical improvement (53).

### Avoidance of Drug Resistance

The World Health Organization (WHO) currently recommends the mass drug administration (MDA) of benzimidazoles (albendazole, mebendazole, pyrantel pamoate, and levamisole) for the treatment and control of soil-transmitted helminths (STH) (54). In 2012, a WHO strategic goal aimed to “eliminate soil-transmitted helminthiases as a public health problem in children” by 2020 (55). In STH-endemic countries, school-aged children were to receive treatment at 75% national coverage and 100% geographical coverage, treating children once or twice annually when STH prevalence is ≥20 and <50 or ≥50%, respectively (56), with a single dose of 400 mg of albendazole. Over 385 million school-aged children at risk of STH received treatment in 2016 alone (68% global coverage), double that of 5 years previously (57). Thirty-eight countries reached their target of 75% coverage (58).

Similarly, the MDA for control of human schistosomiasis aims to prevent morbidity by regular treatment with praziquantel, the only recommended drug for the treatment of human schistosomiasis. In 2018, over 95.3 million people, 87.6% of doses delivered in sub-Saharan Africa, were treated for schistosomiasis (59). Mass treatment is targeted at high-risk groups dependent on the prevalence of infection. Praziquantel is deemed safe in pregnancy, and it is recommended that women, and adolescent girls of child-bearing age, be included in public health interventions.

While the MDA has resulted in considerable progress in the control of STH and schistosomiasis, it has the possibility to drive the potential emergence of AR. While the development and spread of AR in human helminths and the loss of efficacy of albendazole and praziquantel have not yet been confirmed, AR in livestock helminths is widespread (60). Many factors have driven the emergence of AR in animals (Table 3) (61–68), and given the limited drugs available to treat tapeworms in humans, AR remains a potential risk for sustainable chemotherapy, with indefinite rollout of mass chemotherapy.

**Table 3:** Factors influencing the emergence of anthelmintic resistance in livestock.

**Factor**	**Description**	**Livestock**	**Humans**
Treatment frequency	The greater the frequency, the greater the drug pressure and risk for resistance	5–10 treatments per year (61)	1–3 treatments per year (62, 63). Selection for resistance in goats and sheep at these treatment frequencies (64, 65)
Refugia	Proportion of the parasite population free from being exposed to the drug (66)	Can delay emergence of anthelmintic resistance by leaving some animals untreated (1–4% of adult stock) (67)	MDA only targets children, with coverage less than 80%. Adults have the highest worm burdens. Treatment is given during dry seasons, when the proportion of living parasites in the soil is low, for logistical reasons, thereby reducing refugia. As treatment coverage increases for the 2020 goal, the proportion of children treated rises (68).
Under-dosing	Specific dose regimens have different effects on resistance allele frequency, depending on prior frequency of resistance allele in pre-drug parasite population.	Drug efficacy is very high (ERR >99%). Dosing is well-controlled.	Sub-optimal efficacy, never achieve 100% cure. Drugs are often shared among poor families, produced at substandard qualities, and even sold past their expiry date. This could either aid the development of AR (allow for the survival of resistant strains) or delay it (lower drug pressure) (68).

*ERR, egg reduction rate; AR, anthelmintic resistance*.

### Prevention

Prevention of infection is key for success in the management of cysticercosis. Pig-keeping has been promoted as a route out of poverty in LMIC. The large increase in pigs being kept in rural communities has not been matched by efforts to help smallholder farmers keep their pigs healthy. In Tanzania, pig production is one of the fastest-growing livestock sectors, with more than 7% of smallholder famers keeping pigs (69). Pigs are considered by poor farmers in LMIC to require minimum inputs, and farmers see opportunity in keeping free-roaming pigs within the community. To raise healthy pigs, significant veterinary inputs are needed including anthelmintics to break the cycle of *T. solium* transmission between pigs and humans (70). Much can be achieved by cessation of open defecation and penning pigs to prevent them from eating feces, and with health education (70–72). Raising free-range pigs is linked to not only *T. solium* and cysticercosis but also other zoonotic diseases, such as ascariasis and hepatitis E, which trap poor populations in poverty (73).

### Vaccination and Cysticidal Drugs for Pigs

A vaccine for use in pigs against *T. solium*, TSOL18 (Cysvax), has been registered for use in India since 2016 and is undergoing registration in Tanzania, Uganda, South Africa, West Africa, Kenya, Nepal, Philippines, Thailand, and Sri Lanka (73, 74). Cysvax can provide 99.5% protection against porcine cysticercosis, and when combined with the anthelmintic drug, oxfendazole to deworm the pigs, protection can be increased to 99.7% effectiveness (74–76). Importantly, this treatment does not make the pork unfit for human consumption (74, 77, 78). A second vaccine against *T. solium*, SP3VAC, and a modified parenterally administered SP3vac-phage version have undergone trials in Mexico but are yet not available for commercial use. These vaccines all require two doses, and since no vaccine currently treats existing cysts, they would require application of oxfendazole to have “viable cyst”-free pigs (79–81).

### Meat Inspection

Lingual palpation (or tongue inspection) in pigs and meat inspection are used to detect cysticerci in pork, but only around 21% of infected pigs will be detected by lingual examination alone. Depending on infection load and inspection practice, infected pork can easily pass into the food chain. Home-slaughtered pigs and lack of farmer knowledge of the zoonotic risk from *T. solium* heighten and exacerbate the risk in the community. Meester et al. showed that home-slaughtered pigs were 13 times more likely to be contaminated than commercially slaughtered pigs, regardless of the country of origin (82).

Kenya has had a law in place for meat inspection since 1977; all meat must be inspected by ministry officials prior to leaving the slaughterhouse. If cysticerci are found, the pig carcass is condemned, and the meat cannot be sold (83). In one study, inspectors reported that all pigs leaving the slaughterhouse had been inspected; however, no inspectors were visible at the facility, and it was not possible to confirm any inspections having been completed (84). This example shows how contaminated meat can enter the food chain, putting consumers at risk despite legislation being in place.

### Communication and Health Messaging

Most NTDs affect poor people in communities that are poorly served by both medical and veterinary services. Health messaging is challenging in resource-poor communities and particularly challenging for neglected zoonotic diseases. Control tools designed to prevent zoonotic disease transmission for uptake and adoption in the community demand a comprehensive understanding of how the affected community members perceive the disease. This does not necessarily require an explanation of the complex disease causation. Knowledge within communities is also patchy. Communities may erroneously relate epilepsy to witchcraft but correctly associate the presence of white nodules in pigs to bad practice in pig husbandry. Studies in Zambia showed that while some village inhabitants were aware that eating pork containing cysts was unhealthy and could cause disease, other individuals from the same villages saw nothing wrong with eating infected meat, arguing that the cysts gave a satisfying “burst in the mouth feel” (73). Focus group discussions with women in villages endemic for *T. solium* in Zambia showed that the women were aware that pigs brought diseases and worms and especially that pigs ate feces; however, despite this knowledge, pigs were allowed to be predominantly free ranging within the village (73, 85). In contrast, studies in Mozambique showed that only 17.4% of households were aware how pigs acquired the *T. solium* infection (73, 85).

Indirect approaches to prevent open defecation have contributed to community approaches for control of *T. solium* infection. The Community Led Total Sanitation (CLTS) program (https://www.communityledtotalsanitation.org/) focuses on introducing behavioral change, essentially shocking communities into an awareness of fecal contamination in their environment, leading communities to a point where they decide freely that they want to become “Open Defecation Free” (86, 87). The CLTS as a standalone intervention, to prevent pigs from being able to eat human stools in the environment, was not particularly successful in Zambia. There was a significant increase in latrines (31%), but many villages failed to eliminate open defecation practices (86); the study focused on the prevalence of porcine cysticercosis before and after the CLTS implementation but did not address why villages in the study area continued the practice of open defecation. When combined with other interventions, the CLTS is likely to show benefits and is one piece in the elimination toolbox, in addition to improved pig husbandry, training and education programs, vaccines, and MDA. More anthropological studies will be needed to gain a comprehensive understanding of cultural taboos on latrine use and how to make interventions more appealing to communities (86).

For cysticercosis and NCC, educational messages and materials that explain that disease in pigs comes from humans and that preventing pigs from eating human feces can interrupt disease transmission are needed, rather than attempts to explain the complex life cycle and disease epidemiology of *T. solium*. Communication is key, and affected communities have been shown to understand and adopt the message that pigs eat stools and people eat pigs. The computer-based tale of the “The Vicious Worm” (https://theviciousworm.sites.ku.dk/) reinforces this very simple message and has been highly effective with individuals completing the program, achieving an average score of 71% in knowledge, after 1 year of follow-up (88).

### Advocacy

Neglected zoonotic diseases are predominately diseases of the poorest populations, living in close contact with domestic animals, on which they are dependent, in communities often lacking adequate health care for humans and animals (8). In 2014, WHO's 4th annual meeting on Control of Neglected Zoonotic Disease stated that the tools to eliminate cysticercosis were in place; however, no country endemic for cysticercosis has been able to eliminate the disease (89). There has been some progress; in 2000, there were 3,362,000 DALYs for cysticercosis (including NCC), and by 2016, this had reduced to 1,912,000 (90). However, the ambitious targets set within the 2012 WHO NTD roadmap have, unfortunately, not been met.

An elimination study in Peru showed the efficacy of the MDA in both humans and pigs for elimination of taeniasis/cysticercosis/NCC but also showed that 90% coverage was needed in both pigs and humans to prevent transmission (91). The MDA narrative offers policy makers a relatively straightforward solution to a complex zoonotic disease that requires addressing issues of clean water, adequate latrines, and pig husbandry.

Addressing NTDs has contributed to alleviating the human and economic burden they impose on the world's poorest communities. NTD interventions offer one of the best buys in global public health, and NTDs serve as an important indicator for identifying disparities in progress toward both universal health coverage and equitable access to high-quality health services. Against a backdrop of large investments in de-worming for NTDs in humans (schistosomiasis, filariasis, and STH) (38, 52), the lack of advocacy to support the prioritization of vaccination of pigs is of concern if cysticercosis and NCC are to be eliminated. Albendazole and praziquantel have been extensively applied for the MDA programs for NTD control for schistosomiasis and STH (36, 50). Deworm the World (https://www.evidenceaction.org/dewormtheworld/), Children without Worms (http://www.childrenwithoutworms.org), Schistosomiasis Control Initiative (https://schistosomiasiscontrolinitiative.org), and Global Programme to Eliminate Lymphatic Filariasis (GPELF) (https://www.who.int/lymphatic_filariasis/elimination-programme/en/) are just a few of the examples of the many deworming programs being run around the world.

On January 28, 2021, WHO will launch its road map for the NTDs' “Ending the neglect to attain the Sustainable Development Goals: a road map for neglected tropical diseases 2021–2030” (92). The road map sets global targets for 2030 and includes milestones and strategies for prevention, control, elimination, and eradication of 20 diseases and disease groups and cross-cutting targets broadly aligned to the Sustainable Development Goals (SDG's). Taeniasis/cysticercosis has been targeted for control; success is defined as a steady increase in the number of countries with intensified control in hyperendemic areas, increasing from 2 (3%) in 2020 to 4 (6%) in 2023, to 9 (14%) by 2025, and to 17 (27%) by 2030. Cross-cutting targets that will impact on taeniasis/cysticercosis include the target for 100% access to at least basic water supply, sanitation, and hygiene in areas endemic for NTDs and 75% integrated treatment coverage for preventative chemotherapy. The goals also seek to achieve 90% of countries reporting on all their relevant NTDs. By moving from single-disease vertical programs to integrated approaches, it aims to promote improved coordination and collaboration. The overarching 2030 global targets are to reduce by 90% the number of people requiring treatment for NTDs, eliminate at least one NTD in 100 countries, and reduce by 75% the DALYs related to NTDs (90).

Control of taeniasis/cysticercosis/NCC demands a One Health approach from multiple stakeholders, in that the MDA in both humans and pigs, vaccination of pigs, and clean water, latrines, and community education will all be needed to effectively eliminate the infection. *T. solium* transmission dynamics models can contribute to this process including CystiSim and EPICYST (93). For the ambitious goals for 2030 to be met, there is a need for greater understanding of the underlying spatial epidemiology, the socio-economic drivers for pig-keeping, and social, individual, behavioral, and community perception of these neglected infections.

## Author Contributions

CB was responsible for conception, assimilation of works, and drafting of the paper. TB was responsible for examination of anthelmintic resistance. AM and SW were involved in the conception and editing of the manuscript. All authors contributed to the article and approved the submitted version.

## Conflict of Interest

The authors declare that the research was conducted in the absence of any commercial or financial relationships that could be construed as a potential conflict of interest.
